# Profiling of Adipose and Skeletal Muscle in Human Pancreatic Cancer Cachexia Reveals Distinct Gene Profiles with Convergent Pathways

**DOI:** 10.3390/cancers13081975

**Published:** 2021-04-20

**Authors:** Ashok Narasimhan, Xiaoling Zhong, Ernie P. Au, Eugene P. Ceppa, Atilla Nakeeb, Michael G. House, Nicholas J. Zyromski, C. Max Schmidt, Katheryn N. H. Schloss, Daniel E. I. Schloss, Yunlong Liu, Guanglong Jiang, Bradley A. Hancock, Milan Radovich, Joshua K. Kays, Safi Shahda, Marion E. Couch, Leonidas G. Koniaris, Teresa A. Zimmers

**Affiliations:** 1 Department of Surgery, Indiana University School of Medicine, Indianapolis, IN 46202, USA; ashnaras@iu.edu (A.N.); xzhong@iu.edu (X.Z.); au.ernie@gmail.com (E.P.A.); eceppa@iupui.edu (E.P.C.); anakeeb@iupui.edu (A.N.); michouse@iupui.edu (M.G.H.); nzyromsk@iupui.edu (N.J.Z.); maxschmi@iupui.edu (C.M.S.); schlosskn@upmc.edu (K.N.H.S.); schlossde@upmc.edu (D.E.I.S.); hancockb@iupui.edu (B.A.H.); mradovic@iupui.edu (M.R.); joshuakays@gmail.com (J.K.K.); lkoniari@iu.edu (L.G.K.); 2 IUPUI Center for Cachexia Research Innovation and Therapy, Indianapolis, IN 46202, USA; yunliu@iu.edu (Y.L.); safi.shahda@lilly.com (S.S.); marioncouch444@gmail.com (M.E.C.); 3 Department of Biochemistry and Molecular Biology, Indiana University School of Medicine, Indianapolis, IN 46202, USA; 4 Indiana University Simon Cancer Center, Indianapolis, IN 46202, USA; 5 Department of Medical and Molecular Genetics, Indiana University School of Medicine, Indianapolis, IN 46202, USA; ggjiang@iu.edu; 6 Center for Computational Biology and Bioinformatics, Indiana University School of Medicine, Indianapolis, IN 46202, USA; 7 Indiana Center for Musculoskeletal Health, Indianapolis, IN 46202, USA; 8 Department of Medicine, Indiana University School of Medicine, Indianapolis, IN 46202, USA; 9 Department of Otolaryngology—Head & Neck Surgery, Indiana University School of Medicine, Indianapolis, IN 46202, USA; 10 Department of Anatomy, Cell Biology & Physiology, Indiana University School of Medicine, Indianapolis, IN 46202, USA

**Keywords:** pancreatic cancer, pancreatic ductal adenocarcinoma, gene expression, RNAseq, adipose, skeletal muscle, atrophy, cachexia, neoplasia

## Abstract

**Simple Summary:**

More than 80% of patients with pancreatic ductal adenocarcinoma (PDAC) suffer cachexia, characterized by loss of muscle and fat. However, most cachexia studies were predominantly focused on muscle. Our clinical study showed adipose tissue loss as a prognosticator in PDAC cachexia. Our study aims to understand the concurrent muscle and adipose changes using transcriptome profiling. We identified tissue-specific gene expression profiles with changes in adipose being more dynamic. Pathway analysis suggests that muscle and adipose wasting may be mediated through independently targetable mechanisms which may have therapeutic implications. Many of the well-known and novel cachexia genes have been validated using an external muscle and adipose datasets. The study provides the groundwork for future studies to understand if fat wasting precedes muscle wasting in PDAC and if adipose can be targeted for therapeutic interventions. The study also shows that age related muscle loss has distinct mechanisms compared to cachexia.

**Abstract:**

The vast majority of patients with pancreatic ductal adenocarcinoma (PDAC) suffer cachexia. Although cachexia results from concurrent loss of adipose and muscle tissue, most studies focus on muscle alone. Emerging data demonstrate the prognostic value of fat loss in cachexia. Here we sought to identify the muscle and adipose gene profiles and pathways regulated in cachexia. Matched rectus abdominis muscle and subcutaneous adipose tissue were obtained at surgery from patients with benign conditions (*n* = 11) and patients with PDAC (*n* = 24). Self-reported weight loss and body composition measurements defined cachexia status. Gene profiling was done using ion proton sequencing. Results were queried against external datasets for validation. 961 DE genes were identified from muscle and 2000 from adipose tissue, demonstrating greater response of adipose than muscle. In addition to known cachexia genes such as FOXO1, novel genes from muscle, including PPP1R8 and AEN correlated with cancer weight loss. All the adipose correlated genes including SCGN and EDR17 are novel for PDAC cachexia. Pathway analysis demonstrated shared pathways but largely non-overlapping genes in both tissues. Age related muscle loss predominantly had a distinct gene profiles compared to cachexia. This analysis of matched, externally validate gene expression points to novel targets in cachexia.

## 1. Introduction

Cancer associated cachexia is a debilitating multifactorial syndrome characterized by involuntary loss of muscle and fat [1,2,3,4]. Patients with pancreatic ductal adenocarcinoma (PDAC), the major subtype of pancreatic cancer, have a higher proclivity to develop cachexia; such cachexia is significantly associated with PDAC-related deaths [5,6,7,8]. The pathophysiology of PDAC cachexia involves a complex interplay between the host and tumor interactions which results in inflammation, malnutrition, anorexia, and neuroendocrine changes. These complex interactions lead to a series of metabolic changes including Warburg effect resulting in increased Cori cycle, lipolysis and decreased lipogenesis [9,10,11]. Studies using animal models have helped uncover many mechanisms that cause muscle and fat wasting [12,13,14,15,16,17,18], and newer animal models are constantly developed to study PDAC and understand cachexia mechanisms [19,20]. Although most of our understanding about cachexia comes from muscle, it is known that cachexia often involves wasting of fat [4,21,22,23,24]. Fat wasting is prognostic in PDAC cachexia. In one cohort study, patients with PDAC who lost only fat had similarly reduced survival to those with combined fat and muscle loss (10 months less) relative to patients who lose neither, independent of tumor response to therapy [4]. However, the molecular mechanisms of fat wasting in PDAC cachexia are less explored.

Understanding the human biology of cancer cachexia is challenging and datasets to date have been limited. Most human cachexia studies to date have used muscle and fat from multiple cancer types [25]. However, though certain genes may be common across cancer types, it seems likely that there will be both tumor-type and treatment-specific mechanisms. Studying a single cancer type could reduce the heterogeneity and begin to interrogate cachexia in a cancer-specific manner. In human studies as well, the focus has either been muscle or fat but to date, never both.

Our current study aims to address several of these challenges in human PDAC cachexia. The aims of the study are to (i) identify differentially expressed (DE) genes for muscle and adipose from the same individuals, (ii) identify common and unique pathways between muscle and fat in cachexia, (iii) identify tissue specific transcription factors, cytokines, growth factors and receptors, (iv) correlate all profiled genes from muscle and fat to the clinical variable of cancer weight loss grade (CWLG) [2] (v) to validate the differentially expressed (DE) muscle and adipose genes in external datasets and (vi) understand if age related muscle loss has distinct gene signatures when compared to PDAC cachexia. Our study identified differentially expressed, distinct tissue-specific gene expression signatures. Genes within common pathways were differentially present in muscle and adipose. We found genes commonly identified in animal models of cachexia, such as FOXO1, FOXO3, IL6R, ZIP14 and PIK3R1, manifested in two independent muscle human datasets. Inflammation-related pathways emerged as key component from the adipose tissue validation data. Our results also suggest that age related muscle loss had a predominantly distinct gene signatures and regulated pathways when compared to PDAC cachexia indicating that age related muscle loss has distinct molecular mechanisms compared to PDAC cachexia.

This is the first study to perform cachexia-specific muscle and adipose gene profiling from same subjects, the first to report genes associated with fat wasting in PDAC cachexia, and the first to validate both findings in external dataset. We demonstrate that muscle and adipose transcriptomes are well established early in pancreatic cancer and that the adipose response is profound than the muscle response. These distinct, tissue-specific gene expression profiles and pathway modulation suggests that muscle and adipose wasting in cachexia may be mediated through independently targetable mechanisms.

## 2. Results

### 2.1. Study Design and Patient Demographics

**Figure 1:**
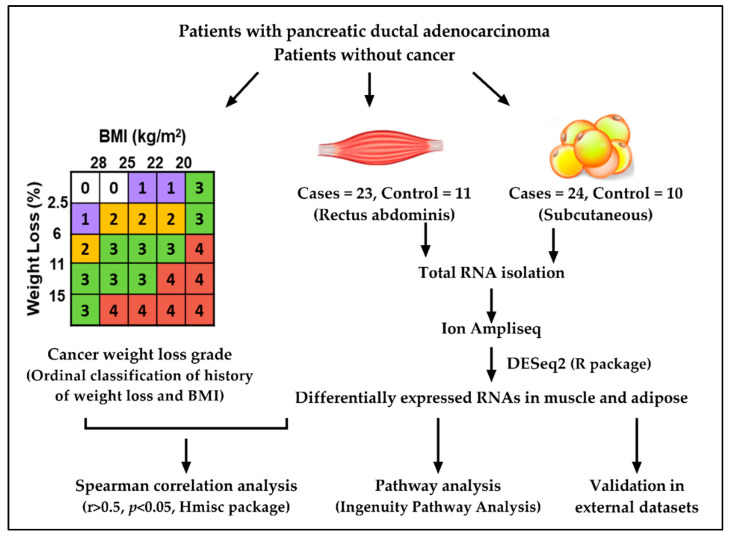
Overall study design.

The overall study design is shown in Figure 1. The summary of patient demographics is presented in Table 1. The mean age of controls and PDAC was different, 50 ± 14 versus 70 ± 11 years (*p* = 0.001). While sex and BMI were not different between groups, weight loss grade was increased in patients versus controls (*p* = 0.005). By CT analysis, skeletal muscle index was significantly less in PDAC patients, while total adipose tissue index was not. However, intramuscular fat was significant between groups and subcutaneous adipose tissue was trending towards significance (Table 1).

### 2.2. Muscle and Adipose Tissue Have Distinct Gene Expression Patterns

**Figure 2:**
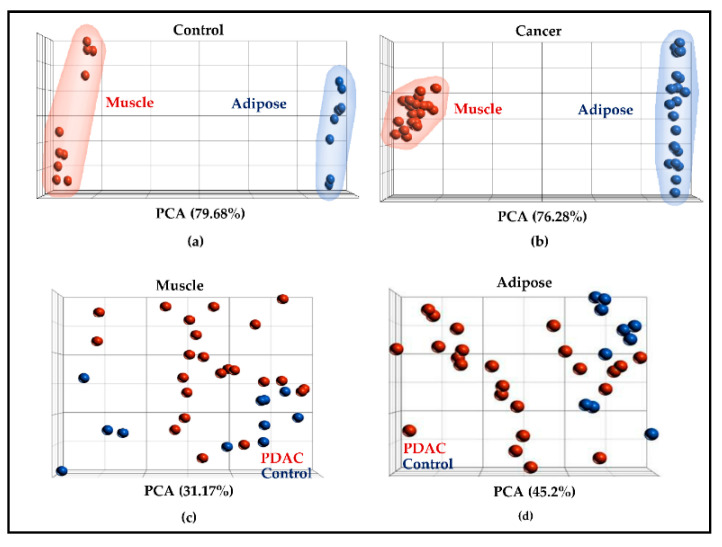
Principal component analysis. All the profiled genes were utilized for the analysis. (**a**,**b**) Indicate that gene expression gene signatures between muscle (**a**) and adipose (**b**) were indeed different in controls and PDAC; (**c**,**d**) illustrate the PCA within muscle (**c**) and adipose in controls and PDAC (**d**).

**Figure 3:**
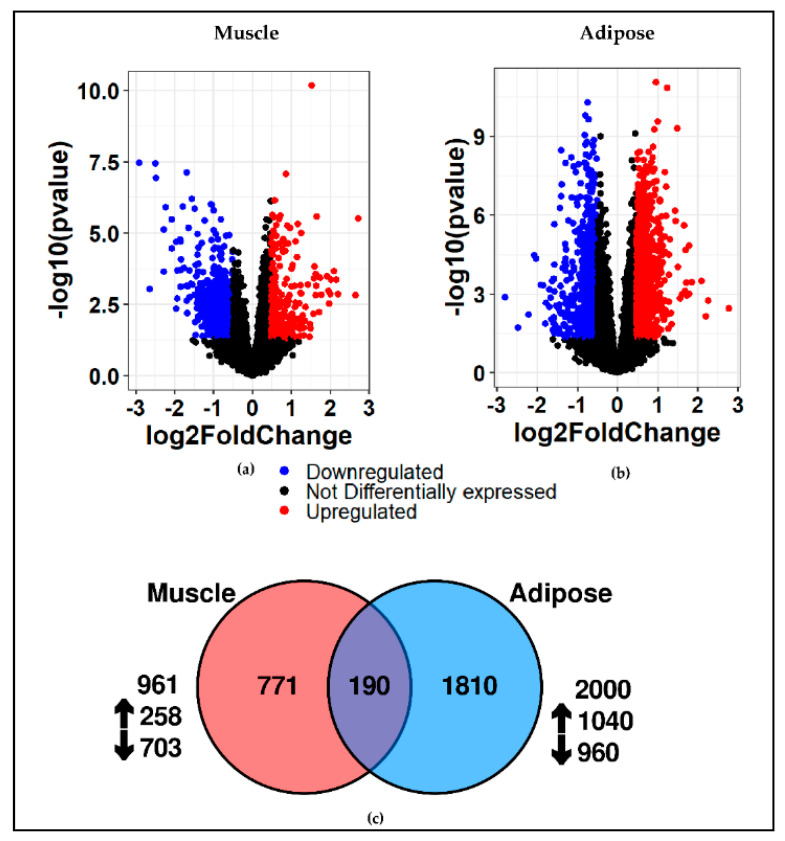
Identification of differentially expressed genes in muscle and adipose. Volcano plot showing the differentially expressed genes for muscle (**a**,**b**) adipose at 1.4 fold change and *p*-value of 0.05. Red indicates upregulation and blue indicates downregulation; (**c**) the common differentially expressed genes between muscle and adipose is ~7%.

In all, 14,177 genes were profiled in muscle and 12,910 genes were profiled in adipose tissue. All the profiled genes served as a starting point for PCA and differential gene expression analysis. As anticipated, PCA showed a clear separation between muscle and adipose genes in controls (Figure 2a) and PDAC (Figure 2b). However, there was no clear separation between controls and PDAC when muscle (Figure 2c) and adipose (Figure 2d) tissues were plotted separately. Differential gene expression analysis between controls and cancer patients identified 961 DE genes in muscle and 2000 DE genes in adipose at a fold change of 1.4 and *p*-value of 0.05. The volcano plot shows the DE genes in muscle (Figure 3a) and adipose (Figure 3b). Overall, 190 genes were common between muscle and adipose which represents a ~7% overlap between the two tissues (Figure 3c), highlighting the tissue specific gene expression patterns. The complete list of DE genes for muscle and adipose is given in Table S1 and Table S2, respectively.

### 2.3. Different Genes Are Involved in Activating or Inhibiting the Common Pathways in Muscle and Adipose

**Figure 4:**
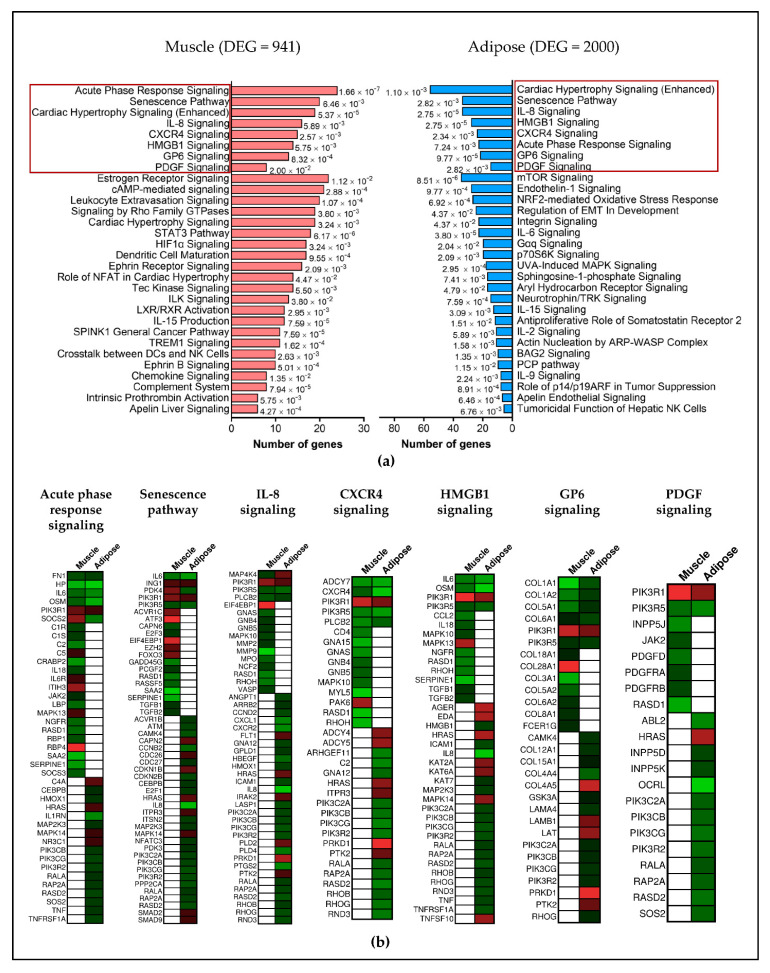
Canonical pathways for muscle and adipose. (**a**) The top pathways highlighted in red indicates the common pathways between muscle and adipose. The unique pathways are also represented in muscle and adipose; (**b**) although there are common pathways between muscle and adipose, the genes involved in activating or inhibiting those pathways are predominantly different, indicating a tissue specific gene expression.

Respectively, 47 and 53 pathways were identified for muscle and adipose (*p* < 0.05 and Z-score > 1.5). Eight pathways were common between muscle and fat: acute phase signaling, senescence pathway, cardiac hypertrophy, IL-8 signaling, CXCR4 signaling, HMGB1 signaling, GP6 signaling and PDGF signaling (Figure 4a). However, the genes involved in activating or inhibiting these common pathways were predominantly different in muscle and adipose tissue (Figure 4b). Other unique pathways identified in muscle include the STAT3 pathway, HIF1α Signaling and LXR/RXR activation (Figure 4a). A few representative pathways that appeared only in adipose tissue include IL-6 signaling, mTOR signaling, Leptin signaling, Oncostatin M signaling and JAK/STAT signaling (Figure 4a). The complete list of pathways, along with the genes involved and *p*-values for muscle is given in Table S3 and for adipose in Table S4.

**Figure 5:**
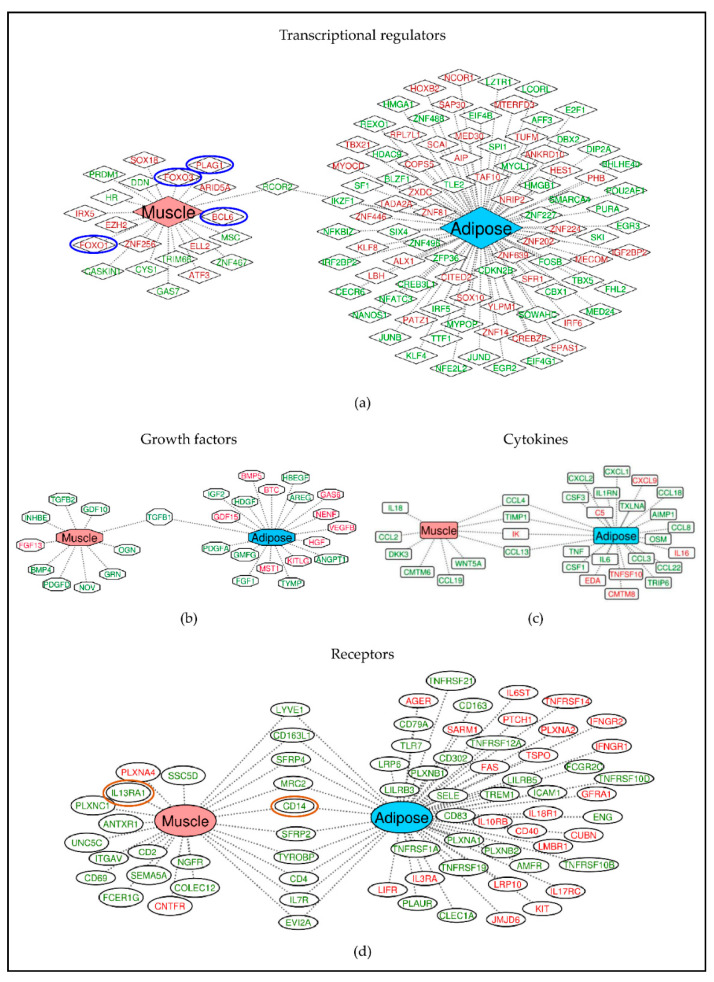
Tissue specific expression of different classes of molecules including transcriptional regulators (**a**), growth factors (**b**), cytokines (**c**) and receptors (**d**). Differentially expressed genes were given as input in IPA and the genes were classified based on their known functions. A strong tissue specific gene expression pattern exists across classes. The genes in red text are upregulated and green text are downregulated genes. The genes represented in between are commonly expressed between muscle and adipose. The genes that are circled in red and blue are also present in aged muscle dataset where the blue circles indicate same direction of effect (upregulated) and red indicates opposite direction of effect (up in one and down in other).

**Figure 6:**
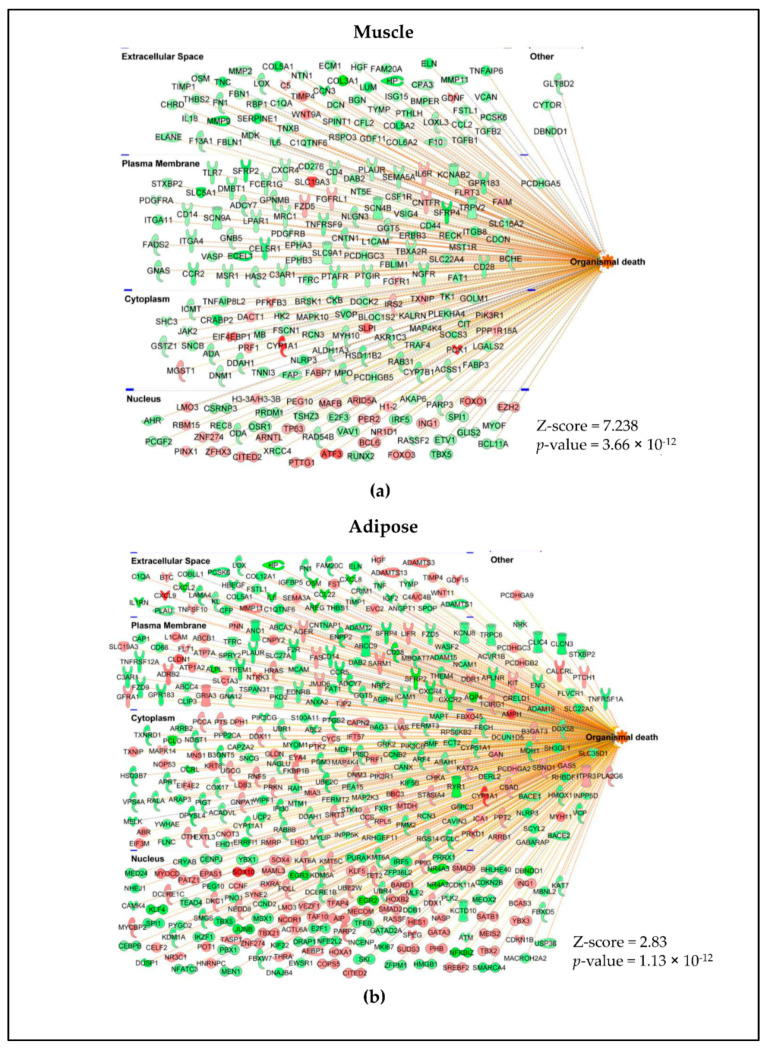
Gene expression predicted organismal death. Genes predicted to enhance (orange lines) or inhibit (blue lines) organismal death versus no prediction (yellow lines) in muscle (**a**) and adipose (**b**).

To further characterize the different classes of molecules that are expressed in muscle and adipose, we classified the DE genes of muscle and adipose into transcriptional regulators (Figure 5a), growth factors (Figure 5b), cytokines (Figure 5c) and receptors (Figure 5d) using IPA. Indeed, many of these genes in all these classes were tissue specific and few were common between muscle and adipose tissue. DE gene profiles were also subjected to disease pathway analysis. The highest association for both muscle (Figure 6a) and adipose (Figure 6b) was “organismal death”.

From these different levels of analyses, it is clear that (i) tissue specific expression patterns exist at gene and pathway levels between adipose and muscle, (ii) adipose tissue gene expression appears to be more dynamic than skeletal muscle in PDAC as the number of adipose genes is approximately twice more than muscle, (iii) inflammation is one of the key drivers of both muscle and adipose wasting, and (iv) the cachexia signature is well-established in muscle and adipose even in patients with early stage, resectable pancreatic cancer.

### 2.4. Correlation of Genes from Muscle and Adipose to Cancer Weight Loss Grade

It is known that combination of weight loss and BMI can differentiate the severity of cachexia and survival [2]. Therefore, to understand whether the genes relate to degree of weight loss, we used all the profiled genes from muscle and adipose and correlated them to cancer weight loss grade, a score generated from BMI and 6-month history of weight loss that has prognostic value in patients with advanced cancers. Only PDAC samples were used for this analysis. To further confirm if these genes are correlated to PDAC alone, we also ran the correlation for controls and removed the genes that were common between PDAC and controls from further interpretation. Genes that correlated with r > 0.5 and *p* < 0.05 were then subjected to clustering analysis (k-means clustering) using the STRING database.

**Figure 7:**
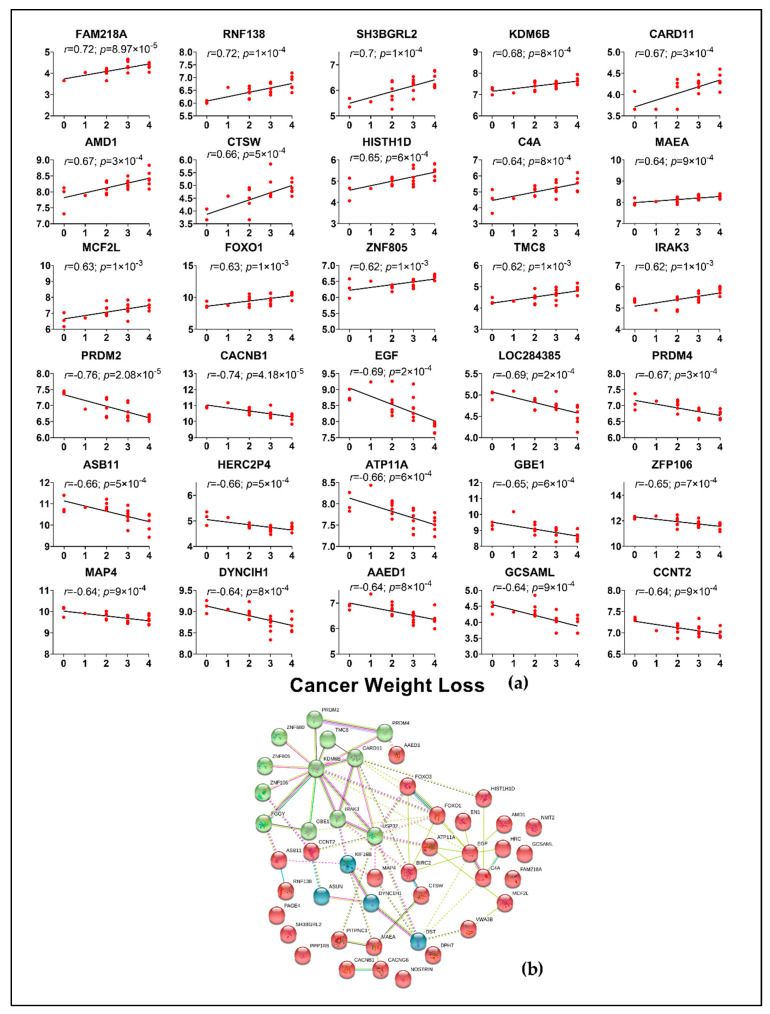
Top 30 muscle genes correlated with CWLG and gene network. (**a**) All genes were correlated against CWLG with PDAC samples alone. Spearman’s rank correlation was performed and only genes with r > 0.5 and *p* < 0.05 were considered. 340 genes correlated with CWLG; (**b**) the network was generated using the STRING database for the top 50 correlated genes with CWLG. Orphan networks were removed.

A total of 340 genes from muscle, including several known genes for cachexia such as FOXO1, FOXO3, PIK3RI, GLUL were correlated with CWLG (Figure 7a and Table S5). Other genes which were not previously implicated such as PPP1R8, WNT9A, SESN1, CCDC68, RNF207, POMT2 and DST were also found to be correlated with CWLG. KDM6B and FOXO1 were identified as top nodal molecules in the network (Figure 7b).

**Figure 8:**
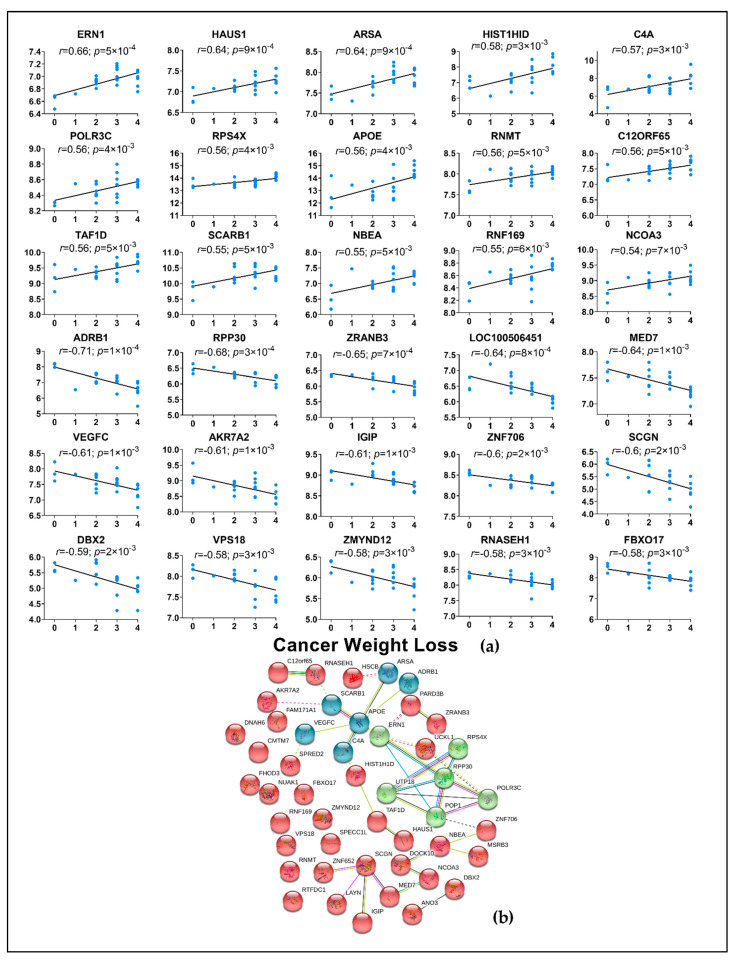
Top 30 adipose genes correlated with CWLG and gene network. (**a**) All genes were correlated against CWLG with PDAC samples alone. Spearman’s rank correlation was performed and only genes with r > 0.5 and *p* < 0.05 were considered and 98 genes correlated with CWLG; (**b**) the network was generated using the STRING database for the top 50 correlated genes with CWLG.

A total of 98 DE genes from adipose tissue were correlated with CWLG and not surprisingly given the relative paucity of data in adipose tissue, the roles of these genes in the context of PDAC and cachexia are undescribed. The top representative genes include RPS4X, AFF3, PDZD8 and DBX2 (Figure 8a and Table S6). APOE was identified as the top molecule in the network (Figure 8b).

### 2.5. Comparison with External Datasets

**Figure 9:**
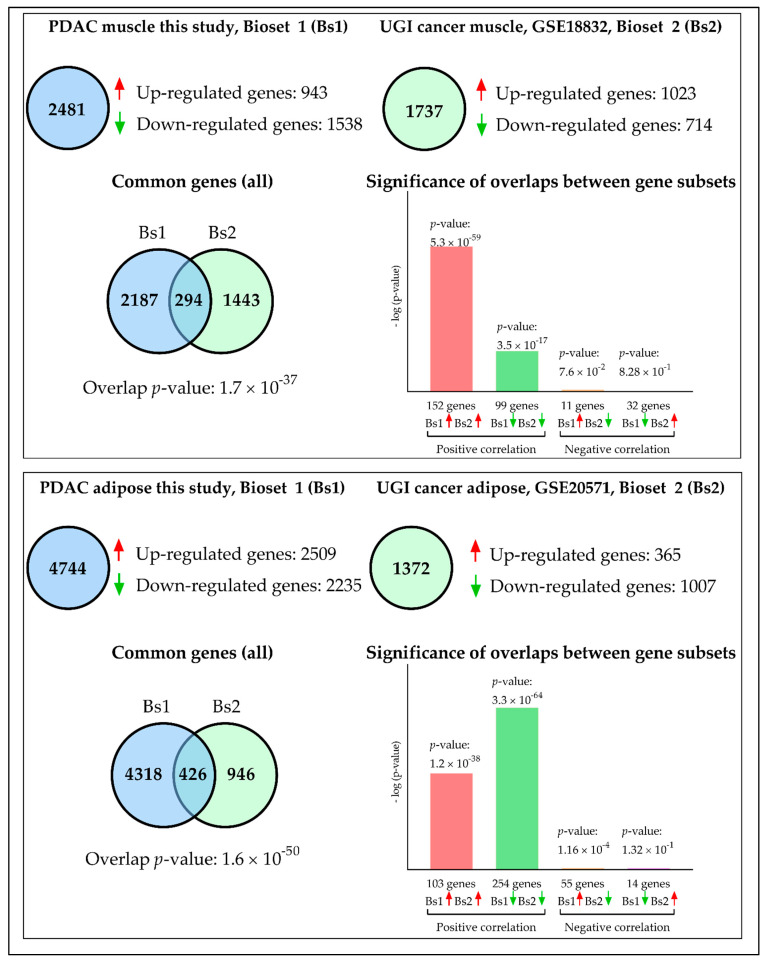
Validation of muscle and adipose DE genes in external dataset. For muscle, 294 genes were common between IU and the external dataset, of which 251 genes had similar direction of effect (~84%). For adipose tissue, 426 genes were common between the two datasets of which 357 genes had similar direction of effect (83%).

We sought to validate our data with external datasets. Few human studies are available with moderate sample size in muscle and only one study has profiled adipose wasting in cachexia to date. Since many of the human studies in cachexia have used array platforms, technological advancement must be considered in deciding the cut-off for the datasets. For example, ion proton sequencing has higher sensitivity and orders of magnitude of detection when compared to microarray [26]. One aim of this analysis was to identify common gene signatures or drivers associated with cachexia across different cancer types. In muscle, 2481 genes were identified in our IU dataset (Table S7), while 1737 genes were identified in the external dataset GSE18832 (Table S8). 294 genes were common between both the datasets, of which 251 genes had similar direction of effect in both datasets (~84%) (Figure 9). In adipose, the IU dataset had 4744 genes (Table S9) while the external dataset GSE20571 had 1372 genes (Table S10). 426 genes were common between both the datasets, of which 357 (~83%) genes showed similar direction of effect in both the datasets (Figure 9).

Interestingly, many functionally validated genes in muscle wasting from model systems and humans such as FOXO1, FOXO3 [27], PDK4 [28], IL6R [29], PIK3R1 [7], LIF [30], and SLC39A14 (also known as ZIP14) [31] were identified in both the human skeletal muscle datasets. The top pathways include regulation of eIF4 and p70S6K signaling, mTOR signaling, and insulin receptor signaling. In adipose, inflammation and its related pathways were highly enriched. Pathways such as acute phase response signaling, IL-8 signaling, IL-10 signaling, complement system, Toll-like receptor signaling, GP6 signaling were identified. The complete lists of common genes and pathways for the muscle and adipose datasets are found in Tables S11 and S12, respectively.

**Figure 10:**
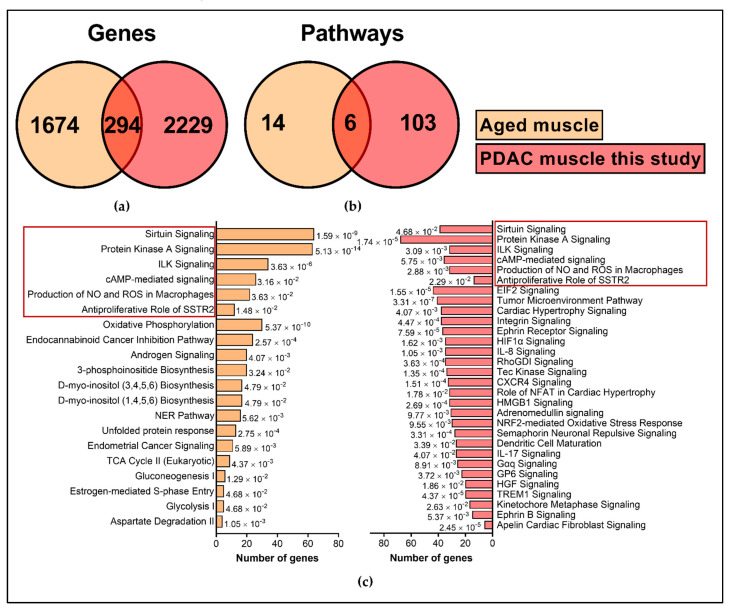
Comparison of age-related transcriptome against PDAC muscle. (**a**) For muscle, 294 genes were common between PDAC muscle (this study) and GSE9676. (**b**) There was a minimal overlap between pathways. (**c**) The list of significant pathways with z-score of 1.5 and *p* < 0.05 are represented for GSE9676 and top 30 pathways in PDAC muscle. The common pathways between the two datasets are highlights in red.

**Figure 11:**
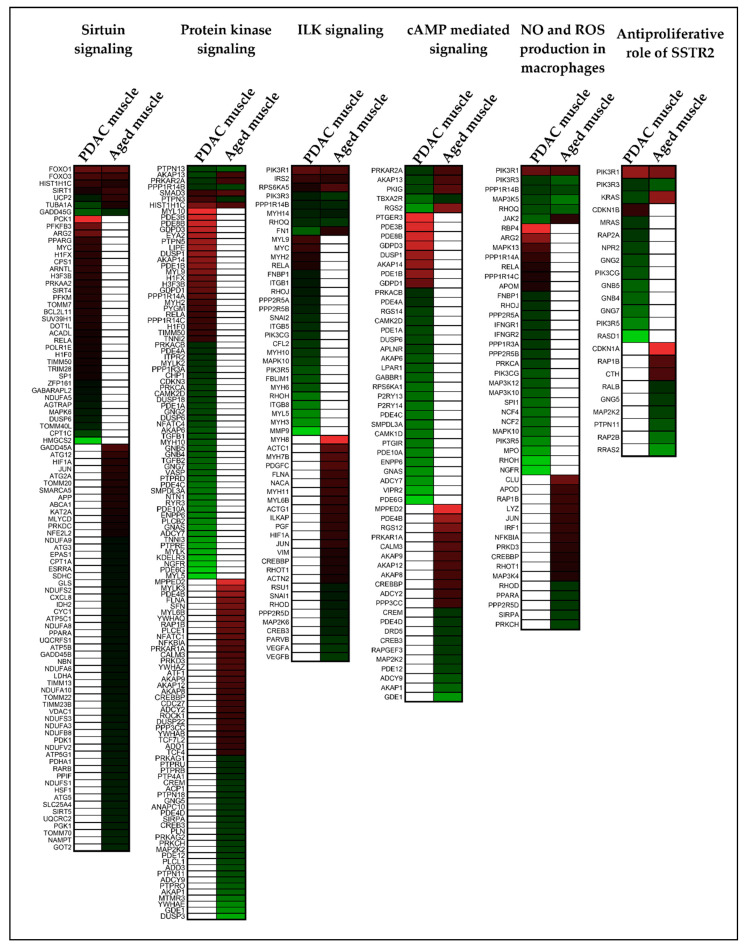
Comparison of age-related transcriptome against PDAC muscle. Six Common pathways were identified between GSE9676 and IU PDAC muscle dataset. Heatmap for each pathway indicate that genes identified in aged muscle dataset and PDAC muscle wasting dataset are predominantly different. Red and green color represent up and downregulated genes, respectively.

It is well documented that gene expression significantly changes with age. As age was significantly different between our control and PDAC, we wanted to understand the extent of gene expression changes common between aging and PDAC. To address this, we identified a non-cancer muscle microarray dataset (GSE9676, GPL 96) which had young (20–30 years old, *n* = 14) and old participants (65–75 years old, *n* = 16) in the study. Only baseline samples were considered for analysis. We considered DE genes with 1.2-fold change and *p* < 0.1 and identified 1968 genes. When overlaid with IU muscle dataset, only 294 genes were common between IU dataset and GSE9676 age dataset (Table S13), while 1674 genes were unique to age and 2229 genes were unique to PDAC muscle (Figure 10). Interestingly, many of the cachexia associated genes such as FOXO1, FOXO3, SIRT1, SMAD3 and FABP3 were identified in age related gene expression in similar direction. Similarly, at the pathway level, only six pathways were common between the two datasets. There was minimal overlap of genes between cachexia and age (Figure 11) suggesting that mechanisms of age-related muscle loss could be strikingly different from cancer related muscle wasting. We did not find any age-related human gene expression dataset for adipose tissue and therefore we restricted the analysis to muscle. The list of significant pathways for PDAC muscle and GSE9676 is given in Table S14 and Table S15, respectively.

## 3. Discussion

This is the first study to report distinct muscle and adipose gene expression profiled from the same patients having same cancer type, PDAC. Although several common pathways were identified between muscle and adipose, the genes involved in these pathways are predominantly different, suggesting that adipose and muscle wasting may be mediated by independent mechanisms. When examining only the patients with cancer, few well known genes and many unknown genes in muscle were found to be highly correlated with degree of cancer weight loss grade. A similar inference cannot be made from the adipose correlation as this is the first study to profile adipose tissue from a homogeneous cohort and the second overall in the literature to profile human genes associated with fat wasting in cachexia. Although the mechanism through which adipose loss occurs in cachexia has been studied in preclinical models [32,33,34,35,36], the specific mechanisms involved in adipose changes in human PDAC cachexia has not been studied till date. We also directed our efforts in validating the signatures in different cancer type from a different geographic location. The fact that some of the well-studied genes in animal models of cachexia are validated in these human datasets is highly promising. Of course, many genes identified in the muscle and adipose validation data point to inflammation, which is a well-established component of cachexia.

As much as the distinct gene expression profiles between muscle and adipose seem obvious, it conveys a critical information that adipose tissue may have independent mechanisms in causing fat wasting providing a potential therapeutic opportunity to target adipose wasting. This was evident from our pathway results where similar pathways had predominantly different and few common genes between adipose and muscle. One possibility for the overall difference in gene expression in adipose versus muscle is that adipose wasting might be well underway prior to overt muscle loss in PDAC cachexia. Although the majority of cachexia research has focused on muscle, our recent clinical study showed that fat loss alone is associated with reduced survival [4]. Furthermore, mechanistic studies in animal models suggest that blocking lipolysis can promote muscle preservation in cachexia [34,37,38]. The molecular mechanisms behind adipose wasting in cancer remain unclear. Our pathway analysis and comparison to the diabesity literature indicate that inflammation could be one of the key drivers of adipose wasting. Indeed, interleukin signaling pathways such as IL-2, IL-6, IL-8, IL-9, IL-15 and IL-23 were observed in adipose. While IL-6 is known to cause fat wasting in cachexia [39], more experimental evidence is needed to understand the interaction between cytokines in augmenting adipose wasting in cachexia. Some of these pro-inflammatory cytokines such as IL-6, IL-8 and IL-23 are implicated in tumor progression and metastasis. IL-4, IL-10 and chemokines recruit circulating monocytes leading to generation of tumor associated macrophages (TAM). The infiltrated monocytes when primed in tumor microenvironment release pro-inflammatory cytokines thereby causing a cachectic environment. However, the exact mechanism through which the macrophages modulate adipose tissue mechanisms in cachexia remains to be elucidated [40]. In another interesting observation, mTOR and NF-kB signaling, which have long been implicated in muscle growth and wasting [41,42,43,44], were identified in adipose tissue here and are known to regulate lipogenesis and lipolysis respectively [45] [46,47]. Based on the IPA, mTOR signaling has a z-score of -2.4, indicating pathway inhibition. The implication of this is unclear, given that the role of mTOR in adipose tissue in cachexia has thus far been little explored.

There is more than one-way that adipose tissue can have a negative impact on skeletal muscle and survival. Evidence suggests that lipids may have an important role in maintaining skeletal muscle mass and on the flip side, it can also affect muscle function. Pathologic accumulation of fat in muscle can cause myosteatosis but the exact mechanisms is not clear, although few genes have been studied in injury model. Increased expression of FABP4, a fatty acid carrier protein leads to accumulation of fat in a muscle injury model [48], and FABP4 is indeed upregulated in our IU PDAC muscle and external datasets. Myosteatosis alone is associated with increased mortality in cancer [49] and Stretch et al., showed that myosteatosis, sarcopenia and the combination are associated with reduced survival in pancreatic and periampullary adenocarcinomas [50]. This suggests that myosteatosis in cachexia affects survival and their molecular mechanisms in cancer cachexia should be delineated. The key conclusions from these findings are: (i) it is critical to study the molecular mechanisms of adipose wasting along with muscle because fat only loss phenotype is possible at least in PDAC cachexia at this point, (ii) mitigating fat loss may also reduce the incidence of myosteatosis thereby improving the overall survival of patients and (iii) many pathways that are extensively studied in muscle wasting but never in adipose wasting were identified and should be investigated further to see if it can lead to newer therapeutic interventions.

For any study, validating the findings in an external dataset adds better value to the existing findings. However, with only a limited number of human studies conducted in cachexia to date, finding well-matched datasets would be ideal but never a reality. The external datasets were profiled using rectus abdominis muscle and subcutaneous adipose tissue similar to our study but were collected from multiple cancer types and using different gene profiling platforms. While the GSE18832 muscle study compared non-cancer controls to cachectic cases as we did, the GSE20571 adipose study compared weight-stable cancer controls to cachectic cases. Given these differences, the common genes may more likely represent true observations of cachexia-specific events. It is most promising that genes such as IL-6R, FOXO1, PDK4, and ZIP14, among others, that have been functionally validated in animal models were identified in both ours and external datasets. Blocking the overexpression of these molecules were shown to reverse muscle wasting and had beneficial effects. Therapeutics targeting FOXO signaling pathways and IL6-R are in various stages of testing in pancreas cancer [51,52], but whether these might have beneficial impact on cachexia needs to be investigated. Given there are many disparities between animal models and human findings in cachexia, findings from human studies must be tested in animal models towards building for a possible trial. To have genes with therapeutic potential expressed in common between humans and model systems augurs well for the future of cachexia research. More efforts are required to understand the mechanisms involved in adipose wasting in cachexia.

In our study cohort, age was significantly different between cases and controls. It is well documented that gene expression changes with age [53,54,55]. To address the influence of age-related gene expression in our study, we analyzed age related muscle gene expression dataset and compared it to our IU PDAC muscle data. Our results showed fewer overlapping genes and more unique genes, suggesting that biological aging process could have different mechanism compared to cancer associated muscle wasting. A similar trend was observed in pathways as well. The key differences in the molecular mechanisms can be attributed to the presence of cancer and its associated chemotherapy effects. It is known that cancer and its related treatment can lead to an accelerated aging process [56]. As well, the effect of chemotherapy is also attributed to accelerated aging as telomere shortening was exacerbated in older patients subjected to chemotherapy [57,58]. One of the common pathways identified was the sirtuin signaling pathway, which has few of the well characterized cachexia genes such as FOXO1 to be present in aging. FOXO family of transcription factors plays an important role in aging and longevity. One of the key roles of FOXO factors is to modulate the ubiquitin proteasome pathway which has been studied in cancer cachexia. In cachexia, activation of FOXO leads to increased activation of ubiquitin ligases leading to proteolysis and eventually muscle wasting [59,60]. FOXO1 was also upregulated in age related gene expression. It could be surmised that FOXO1 could act through the same ubiquitin mechanisms in causing muscle loss in aging which requires further investigation. With loss of muscle mass being central to both aging and cachexia, and with evidence suggesting that inhibition of FOXO transcriptional activity attenuates muscle wasting [27], it could be an interesting target to pursue for therapeutics.

The study has limitations. The differential gene expression between controls and cancer subjects is confounded by the age difference, which we have attempted to address by using an external transcriptome dataset. We will aim to avoid this in our future studies. Another way to address this issue is to perform gene expression between weight stable versus weight losing cancer patients. However, we may not know if the weight stable cancer patients continued to remain weight stable, unless we have a longitudinal follow up of patients, which we lack in this study. As well, in our current study, we have only three weight loss grade 0 patients with PDAC and performing differential expression with such small numbers can lead to compromised results. However, the correlation analysis for cancer weight loss grade were done using cancer patients only and are thus not confounded. Furthermore, we did not analyze the data for sex specific gene differences due to the small sample size. As well, there are different phenotypes observed in cachexia such as muscle only loss, fat only loss or a combination of both, which could not be addressed in this analysis due to the lack of longitudinal body composition data.

## 4. Materials and Methods

### 4.1. Recruitment of Study Subjects

The study was conducted under the Indiana University Institutional Review Board (IRB) approved protocol #1312105608, the same protocol referenced in our prior study [61]. Patients were recruited at Indiana University Hospital between 2014 and 2016. Written and informed consent was obtained. The cancer cohort consists of 24 patients with PDAC-23 had localized PDAC and underwent surgery and 1 participant had chemotherapy prior to surgery. Patients within the control group underwent surgery for non-malignant conditions including hernia repair and cholecystectomy. Rectus abdominis muscle from the incision site and adjacent subcutaneous adipose tissues were collected and snap frozen in liquid nitrogen and stored at −80 °C until further use. In all, we had muscle and adipose tissues from 24 PDAC subjects and 11 non-cancer controls.

### 4.2. Body Composition Measurements Using Computed Tomography

Body composition measurements were obtained from the CT scan obtained as a part of standard of care. The third lumbar vertebrae were used as a landmark to measure the skeletal muscle area (cm^2^), total adipose area (cm^2^) and skeletal muscle density [62,63]. Skeletal muscle index and total adipose index were calculated by normalizing surface area to height (cm^2^/m^2^). Image analysis was performed using the SliceOMatic software. History of weight loss was obtained from patients using the patient-generated subjective global assessment (PG-SGA) [64,65] and confirmed by consulting the medical record. Cancer weight loss grade (CWLG) was calculated using 6-month weight loss history and BMI as described by Martin et al. [2].

### 4.3. Isolation of RNA

RNA was isolated using AllPrep DNA-RNA-miRNA Universal Kit (80224, Qiagen, Valencia, CA, USA). An approximately 10 mg section was removed from the main biopsy in a minimal amount of time. All surfaces were RNase-free. The biopsies were returned to dry ice while the excised piece was homogenized using the TissueRuptor Homogenizer for 30 s in RLT plus buffer. Homogenized lysates were frozen at −80 °C until ready for complete isolation. For muscle, RNA isolation was performed on the Qiacube Instrument using the “Purification of DNA from Tissues or Cells; Part A” and “Purification of RNA, including miRNA, from tissues or cells; Part B” Protocol Sheet. Default elution values were used. RNA was quantified using the RNA-BR Kit and Qubit Fluorometer. For adipose, RNA isolation was performed manually due to the chloroform step in the isolation protocol. Samples were quantified and sized using the Qubit BR RNA Kit and TapeStation RNA Kit.

### 4.4. Library Preparation

For muscle, library preparation was done in accordance with AmpliSeq Transcriptome Human Gene Expression Kit (A26325, Thermo-Fisher Scientific, Waltham, MA, USA, MAN00010742 Rev. A.0) using 10 ng of RNA as starting material. Libraries for all samples were prepared simultaneously and barcoded using Ion Xpress Barcode 1-16 Kit (4471250, Thermo-Fisher Scientific). Libraries were quantified using the Ion TaqMan Quantitation Kit (4468802, Thermo-Fisher Scientific) in Fast Mode using the 7900HT (Thermo-Fisher Scientific). Libraries were diluted to 75 pM for sequencing. For adipose, automated library preparation from 10 ng of RNA was completed according to the Ion Chef protocol (MAN0013432). Libraries were barcoded using the plates available with the Chef Kit and diluted to 50 pM for sequencing. For both tissues, template preparation and sequencing were performed using the Ion PI Hi-Q Chef Kit (A27198, Thermo-Fisher Scientific) with template quantitation using the IonSphere Quality Control Kit (4468656, Thermo-Fisher Scientific).

### 4.5. Differential Gene Expression

Raw reads were obtained by mapping the sequence to the human genome build, hg19. Principal component analysis (PCA) was performed using Partek flow genomic analysis software. DESeq2 was used to identify differentially expressed (DE) genes between control and PDAC using R for both adipose and muscle gene expression analyses [66]. For discovery purposes, a fold change of 1.4 and *p*-value of 0.05 were used to identify DE genes. For PCA and for performing correlation with CWLG, DESeq2-generated variance stabilization counts were used. We refer our dataset as IU datasets which was generated for the study from Indiana University.

### 4.6. Statistical Analysis

Student’s *t*-test was used to compare age, BMI, skeletal muscle index and total adipose index between subject groups. Chi-square test was used for gender comparison and Fisher’s exact test for cancer associated weight loss between non-cancer controls and PDAC cases. Pearson correlation test was used for correlating all the muscle and adipose genes with CWLG. As we wanted to consider only the strong correlations, genes with Pearson r ≥ 0.5 and *p* < 0.05 were selected for downstream analyses. Correlation plots were generated using GraphPad Prism 7. The raw reads and normalized counts for all the samples are deposited in Gene Expression Omnibus database (GSE133979). The overall workflow is presented in Figure 1.

### 4.7. External Validation Datasets

Two datasets from Gene Expression Omnibus data repository were used as contrasts to our own datasets. GSE18832 profiled the rectus abdominis transcriptome of 3 non-cancer controls and 18 upper gastrointestinal cancer subjects via Affymetrix HGU-133plus2 GeneChip array [67]. GSE20571 profiled abdominal subcutaneous adipose tissue of 14 weight stable cancer controls and 13 weight losing cancer patients using Affymetrix Gene 1.0 ST Array [21]. Factoring in the cross-platform differences and discovery intent, we used a relaxed cut-off of 1.2-fold change and *p* < 0.1, for both our dataset and the external dataset for differential gene expression. Datasets were compared using Illumina BaseSpace Correlation Engine [68]. The gene numbers correspond to the output obtained from the correlation engine software.

As age was significantly different between the cases and controls in our study, we wanted to understand if there are any common genes between cachexia and age. We identified a muscle gene expression dataset (GSE9676) profiled using vastus lateralis muscle, that had 14 young and 16 older participants. The “analyze GEO2R” tool in gene expression omnibus database is used for differential expression analysis. We considered only the baseline samples for analysis. There was no human adipose dataset available for age. In line with the other external datasets, we selected differentially expressed genes with 1.2-fold change and *p* < 0.1.

### 4.8. Pathway Analysis for DE Genes and Gene Networks

Ingenuity Pathway Analysis (IPA, Qiagen, Version 2.3, November 2018) was used for pathway discovery, identification of upstream regulators, and classification of genes with fold change >1.4 and *p*-value of 0.05 for both muscle and adipose. Pathways with Z-score > 1.5 and *p* < 0.05 were considered significant. We focused mainly on signaling pathways for muscle and adipose. However, the entire list of pathways is presented in the supplementary information. STRING database version 11.0 was used to generate gene networks for genes that were significantly correlated with CWLG.

## 5. Conclusions

This is the single largest study in cachexia to generate expression profiles of muscle and adipose genes from the same individuals and from the same cancer type to understand the concurrent muscle and adipose wasting in a homogenous population.

## Figures and Tables

**Table 1:** Patient demographics.

Characteristics	PDAC	Non-Cancer Controls	*p*-Value
	*n* = 24	*n* = 11	
Age ^a^	70 ± 11	50 ± 14	0.001
Gender ^b^			N S
Male	12	5	
Female	12	6	
BMI (kg/m^2^) ^a^	28.2 ± 6.5	31.4 ± 6	N S
Weight Loss Grade ^c^			0.005
Grade 0	3	6	
Grade 1	1	3	
Grade 2	6	1	
Grade 3	8	1	
Grade 4	6	-	
Skeletal muscle index (cm^2^/m^2^) ^a^	44.6 ± 10.5	53.2 ± 9.7	0.04
Total adipose index (cm^2^/m^2^) ^a^	235.2 ± 134.2	274 ± 150.8	N S
Intramuscular Fat (cm^2^)	17.65 ± 13.46	10.58 ± 3.90	0.03
Subcutaneous Fat (cm^2^)	226.07 ± 119.09	316.97 ± 110.97	0.055

N S = Not Significant; ^a^ = *t*-test, ^b^ = Chi-square test, ^c^ = Fisher’s exact test. The values are represented as mean ± standard deviation. *p* < 0.05 were considered statistically significant.

## Data Availability

The raw files (bam files) and normalized counts for all the samples are deposited in Gene Expression Omnibus database (GSE133979).

## Supplementary Materials

The following are available online at https://www.mdpi.com/article/10.3390/cancers13081975/s1: Table S1: List of differentially expressed genes in muscle, Table S2: List of Differentially expressed genes in adipose, Table S3: Canonical pathways for muscle using the DE genes, Table S4: Canonical pathways for adipose using the DE genes, Table S5: Complete list of muscle genes correlated with CWLG at r ≥ 0.5 and *p* < 0.05, Table S6: Complete list of adipose genes correlated with CWLG at r ≥ 0.5 and *p* < 0.05, Table S7: IU muscle differentially expressed genes at 1.2 Fold change and *p* < 0.1, Table S8: Differentially expressed muscle genes in external dataset in GSE 18832 at 1.2 Fold change and *p* < 0.1, Table S9: IU adipose differentially expressed genes at 1.2 Fold change and *p* < 0.1, Table S10: Differentially expressed adipose genes in external dataset in GSE 20571 at 1.2 Fold change and *p* < 0.1, Table S11: Canonical pathways for the common genes between IU muscle and external muscle (GSE18832) datasets, Table S12: Canonical pathways for the common genes between IU adipose and external adipose (GSE20571) datasets, Table S13: Differentially expressed age related genes in external dataset in GSE 9676 common to IU muscle dataset at 1.2-fold change and *p* < 0.1, Table S14: Canonical pathways for IU muscle dataset for genes with 1.2 FC and *p* < 0.1, Table S15: Canonical pathways for GSE9676 Old vs young dataset for genes with 1.2 FC and *p* < 0.1.

## Abbreviations

AFF3	AF4/FMR2 Family Member 3
CCDC68	Coiled-Coil Domain Containing 68
DBX2	Developing Brain Homeobox 2
DST	Dystonin
FABP3	Fatty Acid Binding Protein 3
FABP4	Fatty Acid Binding Protein 4
FOXO1	Forkhead Box O1
FOXO3	Forkhead Box O3
GLUL	Glutamate-Ammonia Ligase
IL-10	Interleukin 10
IL-23	Interleukin 23
IL-4	Interleukin 4
IL-6	Interleukin 6
IL6R	Interleukin 6 Receptor
IL-8	Interleukin 8
KDM6B	Lysine Demethylase 6B
LIF	Leukemia Inhibitory Factor
MTOR	Mechanistic Target Of Rapamycin Kinase
NFKB1	Nuclear Factor Kappa B Subunit 1
PDK4	Pyruvate Dehydrogenase Kinase 4
PDZD8	PDZ Domain Containing 8
PIK3R1	Phosphoinositide-3-Kinase Regulatory Subunit 1
POMT2	Protein O-Mannosyltransferase 2
PPP1R8	Protein Phosphatase 1 Regulatory Subunit 8
RNF207	Ring Finger Protein 207
RPS4X	Ribosomal Protein S4 X-Linked
SESN1	Sestrin 1
SIRT1	Sirtuin 1
SLC39A14/ZIP14	Solute Carrier Family 39 Member 14
SMAD3	SMAD Family Member 3
WNT9A	Wnt Family Member 9A
